# High titers of anti-*Leishmania* spp. antibodies in apparently healthy dogs in the North Pioneer Mesoregion of the state of Paraná, Brazil

**DOI:** 10.1590/S1984-29612023023

**Published:** 2023-06-05

**Authors:** Ana Carolina Cavallieri, Débora Sayuri Katto, Luciane Holsback, Eloiza Teles Caldart, Luana Zaboski Pena, Pablo Menegon Castilho, Fernanda Pinheiro Filgueiras, Ellen de Souza Marquez, Mariza Fordellone Rosa Cruz, Kerlei Cristina Médici, Regina Mitsuka-Breganó, Italmar Teodorico Navarro

**Affiliations:** 1 Laboratório de Imunodiagnóstico e Biologia Molecular, Departamento de Ciências Agrárias, Universidade Estadual do Norte do Paraná - UENP, Bandeirantes, PR, Brasil; 2 Laboratório de Zoonoses e Saúde Pública, Departamento de Medicina Veterinária Preventiva, Universidade Estadual de Londrina - UEL, Londrina, PR, Brasil

**Keywords:** Leishmania braziliensis, ELISA, IFAT, cutaneous leishmaniasis, Leishmania braziliensis, ELISA, RIFI, leishmaniose cutânea

## Abstract

Leishmaniasis is an anthropozoonosis with vector transmission, and knowledge regarding the occurrence of this parasitosis in sentinels can contribute to infection and disease control measures in humans. The objectives of this study were to evaluate the occurrence of *Leishmania* exposure and infection in dogs from urban and rural areas in the North Pioneer Mesoregion of the state of Paraná, to evaluate possible risk factors, and to analyze the statistical agreement between the serological techniques that were used. Using a convenience sampling, serum and whole blood samples were collected to perform serological and molecular assays, respectively. The enzyme-linked immunosorbent assay (ELISA) and indirect fluorescent antibody test (IFAT) identified 29/204 (14.2%) and 20/204 (9.8%) seropositive dogs, respectively. Five dogs (2.4%) were seropositive for both serological tests, and four dogs presented high titers in the IFAT. None of the samples tested positive for *Leishmania* spp. DNA according to polymerase chain reaction analysis. No factors were significantly associated with infection. *Leishmania* parasites circulate in urban and rural dogs in the North Pioneer Mesoregion of the state of Paraná. Despite the absence of clinical cases, seropositive animals with high antibody titers should serve as a warning to the local population that should be properly informed regarding the prevention.

## Resumo

A Leishmaniose é uma antropozoonose e sua transmissão ocorre através da picada de flebotomíneos de diferentes gêneros, dependendo da localização geográfica. O conhecimento sobre a ocorrência desta parasitose nos reservatórios pode colaborar com medidas de controle da infecção e doença nos humanos. Os objetivos deste estudo foram: avaliar a ocorrência da infecção por *Leishmania*, em cães de áreas urbanas e rurais, na região da mesorregião Norte Pioneiro do Paraná; avaliar possíveis fatores de risco à infecção e analisar a concordância estatística entre as técnicas sorológicas utilizadas. Para tanto, foram colhidas 204 amostras de soro e sangue total de cães escolhidos aleatoriamente de municípios da mesorregião Norte Pioneiro do Paraná para a detecção de anticorpos anti-*Leishmania* spp., pelo ensaio imunoenzimático (ELISA) e reação de imunofluorescência indireta (RIFI), e para a detecção de DNA de *Leishmania* spp. pela reação em cadeia pela polimerase (PCR). Dos 204 cães testados, 29 (14,2%) e 20 (9,8%) foram soropositivos pelo ELISA e RIFI, respectivamente. Cinco cães (2,4%) foram soropositivos em ambos os testes sorológicos. Não foram observados fatores abióticos e bióticos associados à infecção. A concordância entre as técnicas diagnósticas calculada por Kappa foi de 0,34. Conclui-se que, apesar da baixa ocorrência de positividade para leishmaniose e da ausência de casos sintomáticos, os resultados deste estudo devem ser um alerta para a população local por se tratar de uma importante zoonose e, portanto, medidas de prevenção e controle não devem ser negligenciadas.

## Introduction

Leishmaniasis are major anthropozoonoses caused by several species of protozoa of the genus *Leishmania*, which are transmitted by phlebotomine sand flies and cause visceral leishmaniasis (VL) or cutaneous leishmaniasis (CL) (Noli & Auxilia, 2005). One hypothesis about the possibility that *Leishmania infantum* may circulate in the studied region is that VL is spreading rapidly from cities in the western part of São Paulo state (Presidente Prudente, 205 km from Bandeirantes), which is the most feasible route to be traveled by the disease (parasite and vector) toward the northern border of the Paraná state (D'Andrea et al., 2015).

In Brazil, seven species of *Leishmania* have been identified to cause CL, including *Leishmania braziliensis, Leishmania guyanensis, Leishmania naiffi, Leishmania shawi, Leishmania lainsoni, Leishmania amazonensis,* and *Leishmania lindenbergi* (Gontijo & Carvalho, 2003). *Leishmania (Viannia) braziliensis* is present throughout Brazil from north to the south (Madeira et al., 2003) and is the most prevalent species in Paraná. It causes cutaneous and mucosal lesions. Cases have been reported in domestic dogs with the possibility that they may act as a source of secondary infections. The vectors that have been identified as responsible for the transmission of tegumentary leishmaniasis in the region are *Nyssomyia neivai, Pintomyia pessoai, Migonemya migonei, Nyssomyia whitmani*, and *Pintomyia fischeri*, and all of these transmit *L. braziliensis* (Cruz et al., 2013).

From 2000 to 2019, 208,012 CL cases were reported in Brazil, and 4 (1.9%) cases were reported in the southern region. Of these, 3,780 (92.2%) cases were reported in the state of Paraná (SINAN, 2022). Among CL human cases registered in 268 municipalities of Paraná state between 2001 and 2015, 44.6% of the total were constrained to 10 municipalities that included 7.5% in Londrina, 7.3% in Cianorte, 6.1% in Cerro Azul, 5% in Jussara, 4% in Terra Boa, 3.9% in Bandeirantes, 3.5% in Adrianópolis, 2.8% in Umuarama, 2.4% in Japurá, and 1.9% in Maringá (Melo et al., 2017). The presence of dogs infected with *L. braziliensis* and the notification of cases in humans in the north of the state of Paraná have been associated with altered environments that favor the adaptation of sand fly species and their contact with their hosts and reservoirs (Caldart et al., 2021). The number of human CL cases reported in the period from 2007 to 2019 in the municipalities evaluated in the present study according to the Information System for Notifiable Diseases (SINAN) were as follows: Abatiá (18); Andirá (07); Bandeirantes (160); Cornélio Procópio (01); Itambaracá (03); Joaquim Távora (0); Santo Antônio da Platina (09) (SINAN, 2022).

Cutaneous leishmaniasis can be diagnosed through direct parasitological examination in which the amastigote form is visualized. This test is easy to perform, rapid, and less expensive; however, it is indicated only for cases with skin lesions or lymphadenopathy. The serological test recommended by the Ministry of Health for screening diagnosis is the enzyme-linked immunosorbent assay (ELISA); the indirect fluorescent antibody test (IFAT) should be performed to confirm the serological diagnosis (CRMV-PR, 2015).

Despite several reports of leishmaniasis in humans in the Northern Pioneer Mesoregion of the state of Paraná, there is little information regarding *Leishmania* infection in companion animals in this region. Due to the importance of dogs as sentinels and vectors of *Leishmania*, it is necessary to assess its health status in regard to leishmaniasis in the region. Therefore, the objective of the present study was to determine the presence of anti-*Leishmania* spp. antibodies and *Leishmania* spp. DNA in dogs from urban and rural areas of the Northern Pioneer Mesoregion of Paraná state and to evaluate possible factors associated with *Leishmania* infection.

## Materials and Methods

For convenience, urban and rural dogs from the Northern Pioneer Mesoregion of Paraná were selected. After the invitation and agreement of the guardian to participate in the research, the animal was carefully restrained, and the blood was collected directly from the jugular vein and later aliquoted for packaging in two different sterile tubes that included one without anticoagulant and one with anticoagulant. Blood was sent in polystyrene boxes with recyclable ice to the Laboratory of Immunodiagnosis and Molecular Biology (LIBI) of the State University of Northern Paraná.

An epidemiological questionnaire was applied to the guardians of the animals and contained questions regarding habitat (urban or rural), sex, if the animal left the city or the state of Paraná, if there was complaint of weight loss in recent months, if there was a skin wound that was difficult to heal, if the animal used a repellent collar, if it slept in a sheltered place or outdoors, presence of woods and/or forests near the residence, wild animals in the vicinity, report of the presence of mosquitoes in the home, if there was use or report of use of insecticides near the residence, and whether or not the animal had been vaccinated against leishmaniasis. The analysis to verify the occurrence of statistical significance between the positive animals and epidemiological variables in the questionnaire was performed using the Epi Info program and the Fisher's exact test with a statistical significance level of P<0.05.

Serum and whole blood samples were collected and frozen for subsequent serological and molecular analyses. The ELISA and IFAT assays were performed on all samples that were collected to determine the presence of anti-*Leishmania* spp. IgG antibodies. The preparation of the *L. braziliensis* antigen for sensitization of the ELISA plates was performed according to Szargiki et al. (2009), and the protocol was based on the work of Vicente (2010). To perform the immunoenzymatic assay, four positive controls, four negative controls, one blank, and 39 samples were used in each 96-well microplate. All samples were diluted 1:100 and tested in duplicate. Peroxidase-conjugated anti-antibodies were added at a dilution of 1:5,000. The absorbance was determined immediately using an ELISA reader (iMark, Bio-Rad, Hercules, California, USA) at a wavelength of 490 nm. The cutoff point of each plate was obtained by averaging the optical density (OD) of the negative controls plus three standard deviations. After the calculation per plate, the overall cut-off point was calculated using the receiver operating curve (ROC) using the statistical program MedCalc 13.2.0. The IFAT was performed as described by Oliveira et al. (2008). The slides were prepared with *L. braziliensis* promastigotes, and positive and negative controls were included in all of the slides that were tested. Samples with titers equal to or greater than 40 were considered positive for the presence of anti*-Leishmania* spp. antibodies, and base two dilutions were performed until they were no longer positive. Cohen's kappa coefficient was obtained using the formula K = Po - Pe/1-Pe, where Po=a+d/n and Pe = [(a+b)(a+c)]+[(a+d)(b+d)]/n2, Pe is the expected agreement, Po is the observed agreement, a is a true positive, b is a false positive, c is a false negative, and d is a true negative (Landis & Koch, 1977).

DNA was extracted using the phenol-chloroform-isoamyl alcohol method (Sambrook et al., 1989) using 200 μL of whole blood. After extraction, DNA was eluted in 50 μL of ultrapure water in a refrigerator for 48 h, and samples were then stored at -20 °C until quantification using L-QUANT (Loccus Biotechnology). Only extracts with concentrations of greater than 20 ng/µL were used for polymerase chain reaction (PCR). Samples that did not reach this concentration were subjected to re-extraction, and in case of failure of the second extraction, the sample was discarded from the study. For DNA amplification, nested PCR was performed. Primer oligonucleotides 332 and 221 were used in the first reaction and resulted in a product of 603 base pairs from the 18S rRNA gene. Using this product, a second reaction was performed with primers 333 and 222 to generate fragments of 393 bp as described by van Eys et al. (1992). Ultrapure water was used as a negative control, and DNA from *L. naifii* or *L. mexicana* was used as a positive control.

## Results and Discussion

Reactive samples according to serological diagnosis methods confirm a previous exposure to *Leishmania* antigens. Considering that the study was performed in an endemic region for CL and that all animals were apparently healthy, the results that were obtained corroborate the literature (Távora et al., 2007).

Of the 204 dogs, 29 (14.2%) and 20 (9.8%) were seropositive for *Leishmania* spp. according to ELISA and IFAT, respectively. Studies report that IFAT is considered to be more specific than ELISA, and this could explain the lower positivity of dogs by IFAT in the present study (Szargiki et al., 2009; Oliveira et al., 2006). Five (2.4%) samples were positive for both serological techniques (Table 1) and generated a kappa index that revealed mild agreement (0.34) (Landis & Koch, 1977).

**Table 1:** Distribution of positive and negative results for anti-*Leishmania* spp. antibodies detected using the ELISA and IFAT assays of dogs from the Northern Pioneer Mesoregion of the state of Paraná.

	**IFAT positive**	**IFAT negative**	**Total**
**ELISA positive**	5	24	39
**ELISA negative**	15	160	165
**Total**	20	184	204

Four of the five animals with reagent serum samples in both serological tests exhibited the highest titers in the IFAT study. Animals 43 (A43) and A51 were from the urban area of Joaquim Távora city and exhibited titers of 160. Animals A166 and A116 were from rural areas of Itambaracá and Cornélio Procópio and presented titers of 320 and 640, respectively. None of these animals were of a defined breed, and they had never left the municipality. They had never received any type of vaccine and did not sleep in a sheltered place. Those who lived in rural areas burned garbage and possessed water sources and areas of forest nearby. Residents in urban areas possess vacant land close to the house. Cross-reaction with other parasites such as *Trypanosoma cruzi* is known to occur due to the sharing of epitopes between the protozoa (Luciano et al., 2009); however, in the present study, in addition to positivity in both tests the high titers corroborated a true positive result in these four animals (Proverbio et al., 2014).

In regard to sex, males and females were seropositive by ELISA (12.5% and 15.7%; p=0.6472) and IFAT (10.4% and 9.3%; p=0.9639), respectively, without a statistically significant difference. Similar results were described by Toscano et al. (2013) and were attributed to the knowledge that sandflies perform blood repasses randomly and that the prevalence of disease or infection depends upon the proportion of males and females in each region.

**Figure 1:**
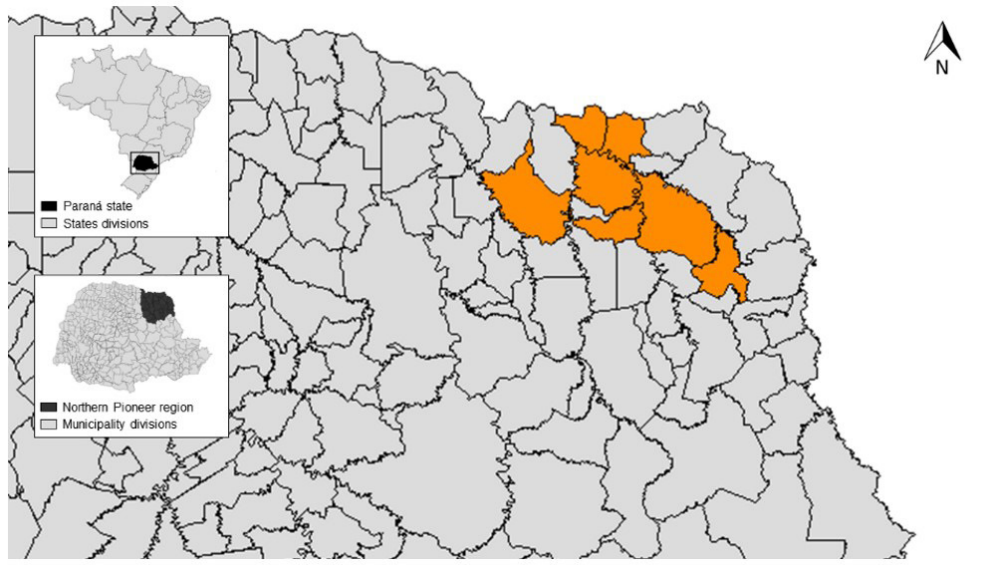
Map of the state of Paraná highlighting the municipalities (orange areas) of residence of dogs in the Northern Pioneer Mesoregion of the state of Paraná. On the top left: Map of Brazil showing the Paraná State (square/black). On the lower left: Map of the Paraná State showing the Northern Pioneer region.

Regarding the origin of the animals, 32 were from Abatiá (15.7%), 30 were from Andirá (14.7%), 35 were from Bandeirantes (17.1%), 26 were from Cornélio Procópio (12.8%), 35 were from Itambaracá (17.1%), 23 were from Joaquim Távora (11.3%), and 23 were from Santo Antônio da Platina (11.3%) (Figure 1). One hundred and ten animals were from rural areas (53.9%), 17 (15.5%) were serologically positive according to ELISA, and nine (8.2%) were positive as assessed by IFAT. Of the 94 urban dogs tested, 12 (12.8%) were seropositive according to ELISA, and 11 (11.7%) were positive according to IFAT with no statistically significant difference (p=0.7321 and p=0.5429, respectively). The results of the serological diagnosis by municipality and area of residence are presented in Table 2.

**Table 2:** Distribution of positive and negative results for anti-*Leishmania* spp. antibodies using the ELISA and IFAT assays in dogs from the Northern Pioneer Mesoregion of the state of Paraná, according to the municipalities and area of residence.

**Municipalities**	**ELISA**				**IFAT**			
	**Urban**		**Rural**		**Urban**		**Rural**	
	**n/N**	**(%)**	**n/N**	**(%)**	**n/N**	**(%)**	**n/N**	**(%)**
**Abatiá**	1/14	7.1	2/18	11.1	1/14	7.1	0/18	0.0
**Andirá**	0/14	0.0	3/16	18.8	3/14	21.4	0/16	0.0
**Bandeirantes**	5/18	27.8	5/17	29.4	1/18	5.5	3/17	17.6
**Cornélio Procópio**	1/11	9.1	1/15	6.7	0/11	0.0	2/15	13.3
**Itambaracá**	0/14	0.0	3/21	14.3	2/14	14.3	1/21	4.8
**Joaquim Távora**	1/11	9.1	1/12	8.3	2/11	18.2	1/12	8.3
**Santo Antônio da Platina**	4/12	33.3	2/11	18.2	2/12	16.7	2/11	18.2
**Total**	**12/94**	**12.8**	**17/110**	**15.5**	**11/94**	**11.7**	**9/110**	**8.2**

In the analysis of the clinical and environmental variables of the epidemiological questionnaire, there were no statistically significant differences (P>0.05) (Table 3). None of the 204 dogs that were evaluated had been vaccinated against leishmaniasis or used a repellent collar. The absence of vaccination was expected, as it is indicated mainly for endemic regions for VL; however, the use of the collar is recommended due to the endemic status of CL (Lopes et al., 2018). Guardians whose animals were positive were advised to seek veterinary medical assistance. Additionally, they were instructed to take the precautions recommended by the Ministry of Health and the Federal Council of Veterinary Medicine.

**Table 3:** Observed frequencies of the variables of the epidemiological questionnaire according to the diagnostic tests used in samples of dogs from the North Pioneer region of the state of Paraná.

**Variables**	**ELISA**		**IFAT**	
	**n/N**	**(%)**	**n/N**	**(%)**
***Home area***				
Rural	17/110	15.5	9/110	8.2
Urban	12/94	12.8	11/94	11.7
***Gender***				
Male	12/96	12.5	10/96	10.4
Female	17/108	15.7	10/108	9.3
***Has the animal moved out of its birthplace?***				
Yes	8/60	13.3	8/60	13.3
No	17/119	14.3	11/120	9.2
Don't know (adopted animal)	4/25	16.0	1/25	4.0
***Has the animal shown weight loss in the last 90 days?***				
Yes	3/35	8.6	2/35	5.7
No	26/169	15.4	18/169	10.7
***Does the animal have any skin sores?***				
Yes	3/23	13.0	2/23	8.7
No	26/181	14.4	18/181	9.9
***Does the animal spend the night in a sheltered place?***				
Yes	0/16	0.0	3/16	18.7
No	28/180	15.6	17/180	9.4
Sometimes	1/8	12.5	0/8	0.0
***Are there rivers, streams, dams or lakes near the residence/property?***				
Yes	23/146	15.7	13/146	8.9
No	6/58	10.3	7/58	12.1
***Do you have woods, forests or valley bottoms near the residence/property?***				
Yes	24/147	16.3	14/146	9.6
No	5/57	8.8	6/58	10.3
***Do you have mosquitoes in your house?***				
Yes, many	15/95	15.8	11/95	11.6
Yes, few	11/91	12.1	7/91	7.7
No	3/18	16.6	2/18	11.1
***Are pesticides used near the property?***				
Yes	19/107	17.7	9/107	8.4
No	10/97	10.3	11/97	11.3

All of the whole blood samples that were evaluated were negative for *Leishmania* spp. DNA. The animals selected for this study exhibited no clinical signs, and therefore it is possible that all of the individuals that were collected were not infected with *Leishmania* spp. or were not in parasitemia. Using lymph nodes (Reale et al., 1999) and skin (Manna et al., 2004) aspirated samples is more suitable than is using whole blood for *Leishmania* spp. DNA amplification due to the higher number of parasites (Lombardo et al., 2012). Therefore, for the diagnosis of leishmaniasis it is important to combine several diagnostic methods to allow for greater diagnostic accuracy (Motta et al., 2021).

## Conclusion

This study concluded that parasites of the genus *Leishmania* circulate in urban and rural dogs in the Pioneer North region of Paraná. Despite the absence of clinical cases, seropositive animals with high antibody titers should serve as a warning to the local population that should be properly informed regarding the prevention and control measures recommended by competent institutions.

## References

[B001] Caldart ET, Sevá AP, Pinto-Ferreira F, Paschoal ATP, Oliveira JS, Cortela IB (2021). American cutaneous leishmaniasis associated with degradation of native forest, regardless of economic, social and infrastructure vulnerability. Zoonoses Public Health.

[B002] CRMV-PR (2015). Manual técnico: leishmanioses caninas.

[B003] Cruz CFR, Cruz MFR, Galati EAB (2013). Sandflies (Diptera: Psychodidae) in rural and urban environments in an endemic area of cutaneous leishmaniosis in southern Brazil. Mem Inst Oswaldo Cruz.

[B004] D’Andrea LAZ, Fonseca ES, Prestes-Carneiro LE, Guimarães RB, Yamashita RC, Soares CN (2015). The shadows of a ghost: a survey of canine leishmaniasis in Presidente Prudente and its spatial dispersion in the western region of São Paulo state, an emerging focus of visceral leishmaniasis in Brazil. BMC Vet Res.

[B005] Gontijo B, Carvalho MLR (2003). Leishmaniose tegumentar americana. Rev Soc Bras Med Trop.

[B006] Landis JR, Koch GG (1977). The measurement of observer agreement for categorical data. Biometrics.

[B007] Lombardo G, Pennisi MG, Lupo T, Migliazzo A, Caprì A, Solano-Gallego L (2012). Detection of *Leishmania infantum* DNA by real-time PCR in canine oral and conjunctival swabs and comparison with other diagnostic techniques. Vet Parasitol.

[B008] Lopes EG, Sevá AP, Ferreira F, Nunes CM, Keid LB, Hiramoto RM (2018). Vaccine effectiveness and use of collar impregnated with insecticide for reducing incidence of *Leishmania* infection in dogs in an endemic region for visceral leishmaniasis, in Brazil. Epidemiol Infect.

[B009] Luciano RM, Lucheis SB, Troncarelli MZ, Luciano DM, Langoni H (2009). Avaliação de reatividade cruzada entre antígenos de *Leishmania* spp. e *Trypanosoma cruzi* na resposta sorológica de cães pela técnica de imunofluorescência indireta (RIFI). Braz J Vet Res Anim Sci.

[B010] Madeira MF, Uchôa CMA, Leal CA, Silva RMM, Duarte R, Magalhães CM (2003). *Leishmania (Viannia) braziliensis* em cães naturalmente infectados. Rev Soc Bras Med Trop.

[B011] Manna L, Vitale F, Reale S, Caracappa S, Pavone LM, Morte RD (2004). Comparison of different tissue sampling for PCR-based diagnosis and follow-up of canine visceral leishmaniosis. Vet Parasitol.

[B012] Melo HA, Rossoni DF, Teodoro U (2017). Spatial distribution of cutaneous leishmaniasis in the state of Paraná, Brazil. PLoS One.

[B013] Motta LM, Ebert KG, Batista KZS (2021). Diagnóstico imunológico e molecular da leishmaniose visceral canina: revisão. Pubvet.

[B014] Noli C, Auxilia ST (2005). Treatment of canine Old World visceral leishmaniasis: a systematic review. Vet Dermatol.

[B015] Oliveira LS, Julião FS, Souza VMM, Freitas DS, Souza BMPS, Paule BJA (2006). A utilização da imunofluorescência indireta no diagnóstico de rotina da leishmaniose visceral canina e suas implicações no controle da doença. Ciênc Anim Bras.

[B016] Oliveira TMFS, Furuta PI, Carvalho D, Machado RZ (2008). A study of cross-reactivity in serum samples from dogs positive for *Leishmania* sp., *Babesia canis* and *Ehrlichia canis* in enzyme-linked immunosorbent assay and indirect fluorescent antibody test. Rev Bras Parasitol Vet.

[B017] Proverbio D, Spada E, Giorgi GB, Perego R, Valena E (2014). Relationship between *Leishmania* IFAT titer and clinicopathological manifestations (clinical score) in dogs. BioMed Res Int.

[B018] Reale S, Maxia L, Vitale F, Glorioso NS, Caracappa S, Vesco G (1999). Detection of *Leishmania infantum* in dogs by PCR with lymph node aspirates and blood. J Clin Microbiol.

[B019] Sambrook J, Fritsch EF, Maniatis T (1989). Molecular cloning: a laboratory manual.

[B020] SINAN (2022). O Sinan.

[B021] Szargiki R, Castro EA, Luz E, Kowalthuk W, Machado AM, Thomaz-Soccol V (2009). Comparison of serological and parasitological methods for cutaneous leishmaniasis diagnosis in the state of Paraná, Brazil. Braz J Infect Dis.

[B022] Távora MPF, Pereira MAVC, Silva VL, Vita GF (2007). Estudo de validação comparativo entre as técnicas ELISA e RIFI para diagnosticar *Leishmania* sp. em cães errantes apreendidos no município de Campos dos Goytacazes, estado do Rio de Janeiro. Rev Soc Bras Med Trop.

[B023] Toscano CP, Rossi CN, Ribeiro VM, Laurenti MD, Larsson CE (2013). Caracterização clínica e epidemiológica das leishmanioses em cães no estado de São Paulo. Braz J Vet Res Anim Sci.

[B024] van Eys GJJM, Schoone GJ, Kroon NCM, Ebeling SB (1992). Sequence analysis of small subunit ribosomal RNA genes and its use for detection and identification of *Leishmania* parasites. Mol Biochem Parasitol.

[B025] Vicente LS (2010). Leishmaniose felina e sua associação com imunodeficiência viral e toxoplasmose em gatos provenientes de área endêmica para leishmaniose visceral.

